# Cord blood lead screening in Lebanon: findings from the environmental exposures in Lebanese infants (EELI) study

**DOI:** 10.1080/26395940.2025.2524084

**Published:** 2025-07-10

**Authors:** Emile Whaibeh, Bassam Eid, Jowy Abi Hanna, Norma Aouad, Joyce Haddad, Georges Abi Tayeh, Myriam Mrad

**Affiliations:** a Institut Supérieur de Santé Publique, Faculté de médecine, Université Saint-Joseph, Beirut, Lebanon;; b Public Health Department, Faculty of Health Sciences, University of Balamand, Beirut, Lebanon;; c Department of Mother and Child Health, Health and Environment Response Agency (HERA), Beirut, Lebanon;; d Hôtel-Dieu de France University Hospital, Beirut, Lebanon;; e Directorate of Preventive Health Care, Ministry of Public Health, Beirut, Lebanon;; f Saint Joseph Fertility Center, Beirut, Lebanon

**Keywords:** Lead exposure, cord blood, pregnant women, Lebanon, heavy metals

## Abstract

This study aims to assess cord blood levels among pregnant women in Lebanon as part of the EELI study. Seventy-five umbilical cord blood samples collected from 2021 to 2022 were analyzed for lead content. Bivariate and multivariate tests were conducted to explore associations and identify predictors of elevated lead levels. The median lead concentration in cord blood was 0.545 μg/dL (range: 0.238 – 5.44 μg/dL). Higher maternal pre-pregnancy BMI and active smoking during pregnancy were associated with elevated lead levels (OR = 2.94, *p* = 0.005; OR = 14.94, *p* = 0.035). Surprisingly, proximity to construction sites was associated with lower lead levels (OR = 0.13, *p* = 0.037), possibly due to safer, modern infrastructure in newly developed areas with reduced legacy contamination. The findings indicate the need for continuous monitoring and public health interventions. While the sample size limits the generalizability of the findings, this pilot study offers important preliminary insights and underscores the need for large-scale screening, and awareness-raising campaigns.

## 1. Introduction

Lead is a hazardous heavy metal prevalent in the environment. Exposure to lead can have life-threatening consequences and long-term negative health impacts on both health and the environment. Exposure to lead alone is estimated to account for 21.7 million disability-adjusted life years (DALYs) worldwide due to long-term health effects, including 30% of the global burden of idiopathic intellectual disability, 4.6% of the global burden of cardiovascular diseases, and 3% of the global burden of chronic kidney diseases [1]. Because bones store lead for decades, even low exposure to lead can result in lifelong health issues, particularly for vulnerable populations such as children, pregnant women, and construction workers [2,3].

In the Middle East and North Africa (MENA) region, lead exposure remains a public health threat due to environmental deterioration, rapid urbanization, and insufficient regulations. A Global Burden of Disease analysis by Rezaee et al. [4] showed that while the burden of lead exposure has declined globally, MENA countries are still experiencing disproportionately high rates of lead-related disability-adjusted life years. In 2019, lead exposure accounted for 414.2 DALYs per 100,000 and chronic kidney disease for 28.7 DALYS per 100,000. The most common sources of lead in the area, according to a systematic study by Al Sukaiti et al. [5], are occupational exposure, followed by traditional cosmetics like kohl and residential sources such contaminated dust, paint, and water. Moreover, important contributions are also made by urban air pollution, with significant lead concentrations found in particulate matter close to industrial and heavy traffic zones [6,7]. Despite the risks, the region is still under-monitored with many gaps in reliable exposure data and limited environmental and public health records [5].

Despite its documented risks, screening for heavy metals, including lead, in Lebanon is largely absent, and childhood lead poisoning in Lebanon is not considered a public health priority. A study conducted by Nuwayhid et al. [8], which collected samples between 1997 and 1998, found that 14% of a sample of Lebanese children aged 1–3 had blood lead levels greater than 10 μg/dL – a level known to cause harmful health effects. That study was conducted when leaded gasoline was widely used in Lebanon, with nearly 90% of automobiles running on leaded fuel. Since then, in an important public health measure, the Lebanese parliament passed Law 341/2002 in 2001, which banned the use of leaded gasoline in Lebanon as of 1 January 2002 [2]. However, there has been little follow-up or sustained effort to monitor and mitigate lead exposure from other sources such as paint, ceramics, jewelry, cosmetics, plumbing, and industrial products continues to be unregulated.

To address this gap, under the patronage of the Lebanese Ministry of Public Health and in Collaboration with the World Health Organization (WHO), the [9] launched from the 22nd to the 28th of October 2023, the ‘End Childhood Lead Poisoning’ National Campaign, under the slogan ‘Let us Lead the Change’ (HERA, 2023). The campaign aimed to take action to eliminate and reduce lead exposure from all sources and to advocate for a healthier, lead-free world. The campaign was part of the ongoing efforts of the Global Alliance to Eliminate Lead in Paint, a joint initiative led by the UN Environment Programme (UNEP) and the WHO.

Building on the momentum of the campaign, with the support of the World Health Organization, the current study was conducted to examine heavy metal blood levels among a sensitive group, pregnant women enrolled in the Environmental Exposure in Lebanese Infants (EELI) study [10]. The pilot aims to assess current cord blood lead levels to inform future public health recommendations, including needed interventions and policy changes to safeguard vulnerable populations from chemical exposures in Lebanon.

## 2. Methodology

### 2.1. Cohort description

The current study utilizes umbilical cord blood samples collected as part of an ongoing birth cohort, the EELI study, launched in 2021, as a collaboration between the University of Balamand (UoB) and Saint-Joseph University of Beirut (USJ). [10]. Since its launch, the study has recruited *n* = 135 pregnant women at Hôtel-Dieu de France University Hospital, recording over 500 variables per participant and biobanking more than 1,000 biological specimens for analysis. However, for the current analysis, only 75 cord blood whole blood samples were analyzed for lead due to funding constraints in this pilot phase as well as the availability of sufficient biospecimen volume meeting quality assurance standards. The samples were collected between 2021 and 2022, and stored at −80°C, following standardized protocols to minimize the contamination risks and to maintain the integrity of the samples. Each sample consisted of an aliquot of 0.5 mL, collected originally in heparin tubes and then transferred into cryovials. Sociodemographic variables and environmental exposure variables, such as proximity to construction sites, factories, agricultural lands, and traffic, were self-reported by participants via surveys. Ethical approval for the study and informed consent procedures are detailed in the Statements and Declarations section of the manuscript.

### 2.2. Laboratory analysis

In collaboration with a trusted sub-contracted courier, samples were securely transported to a designated laboratory for analysis at RECETOX laboratories in the Czech Republic. Adherence to transportation regulations and paperwork was ensured. RECETOX, chosen for its expertise in monitoring toxic compounds in the environment and human tissues, conducted the cord blood lead analysis and communicated the results in μg/dL with the EELI study research team.

### 2.3. Statistical analysis

Statistical Analyses were performed using STATA. Categorical variables were presented as frequency tables, and continuous variables were presented as means and standard deviations. The participants were stratified into two groups according to the median umbilical cord blood lead level. Associations between the stratified cord blood lead level groups and categorical variables were assessed using chi-square and Fisher’s exact tests. A p-value of <0.05 was considered statistically significant.

## 3. Results

### 3.1. Demographic characteristics

Table 1 shows the demographic characteristics of the study sample. The participants had a mean age of 31.9 years (SD = 3.91), ranging from 24 to 42 years. Regarding education, 16.67% of women and 22.22% of their spouses had a high school education or less, while the majority held a bachelor’s degree or higher. Employment status showed that 76.39% of women and 95.83% of their spouses were employed, with a significant portion of women and men working full-time and being self-employed. Household income varied, with nearly half (48.61%) earning over 3400 USD monthly. The main breadwinner was evenly split between husbands and a 50–50 contribution. The MacArthur Subjective Social Status Scores indicate moderate perceived social status both within their own communities and nationally.

### 3.2. Umbilical cord blood lead levels

**Figure 1.**
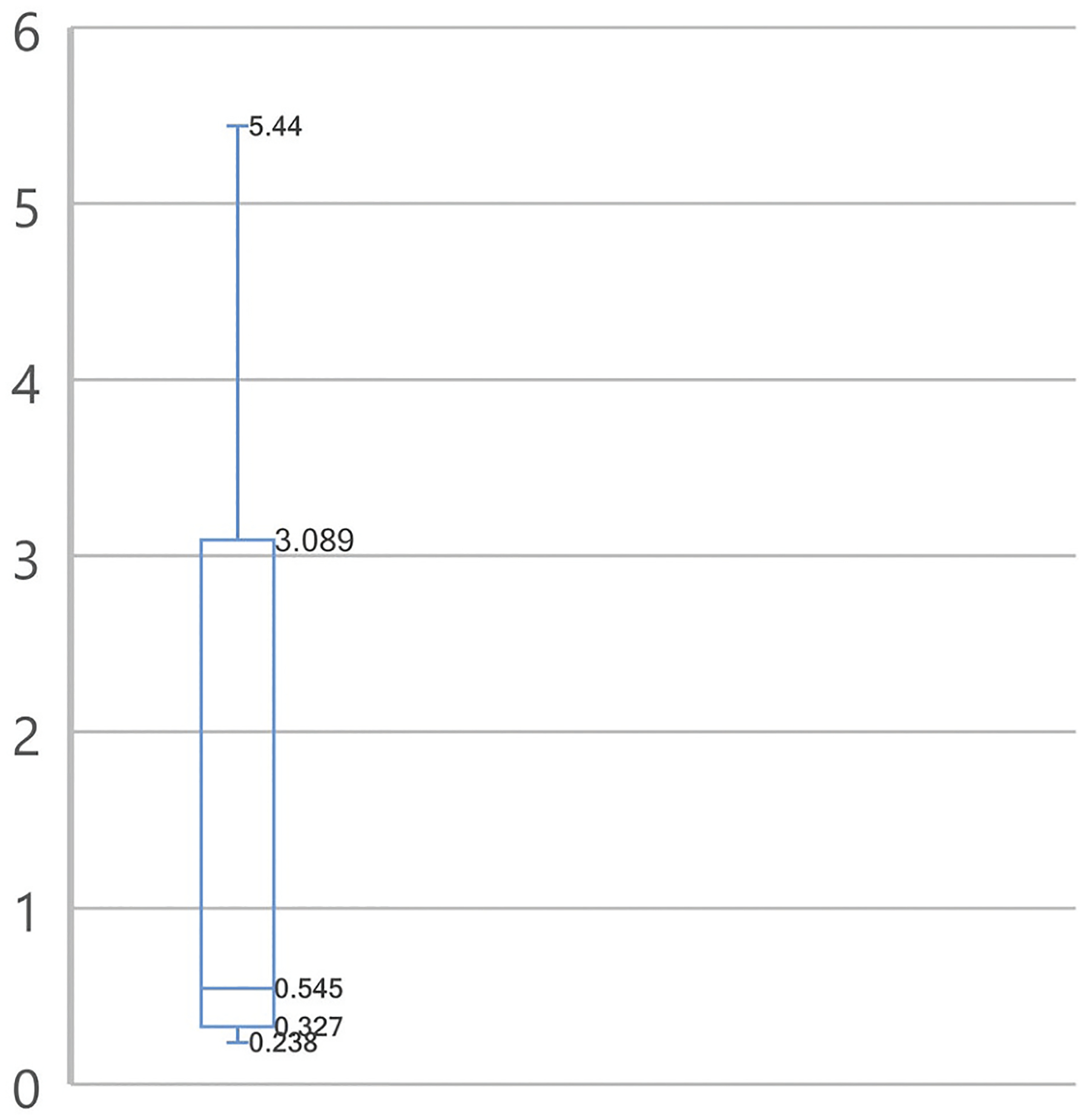
Box and whiskers plot, showing the cord blood lead levels (in μg/dl) among the study participants (*N*=75). The median value was 0.545 μg/dL.

The median concentration of cord blood lead was 0.545 μg/dL (mean: 0.719 ± 0.73 μg/dL) and ranged between 0.238 μg/dL and 5.44 μg/dL, as shown in Figure 1. The 25th and 75th percentiles were 0.416 μg/dL and 0.738 μg/dL, respectively. Lead was detected in all 75 cord blood samples; however, among the analyzed samples, only 1 sample (cord blood level = 5.44 μg/dL) had umbilical cord blood lead levels above the United States Center for Disease Control and Prevention (CDC) blood lead reference value of 3.5 μg/dL, which reflects the 97.5th percentile of blood lead distribution in U.S. children aged 1–5 years from the National Health and Nutrition Examination Survey (NHANES) data [11].

### 3.3. Association between umbilical cord blood levels and socio-demographic and environmental factors

The analysis of the participants’ socio-demographic characteristics (Table 2) and environmental variables (Table 3), stratified by blood lead levels compared to the median, showed significant associations with the mother’s age (p = 0.025) and the house location (p = 0.031). No other variables, including education level, employment type, employment sector, income, and proximity to various sources of pollution, showed any statistically significant associations with umbilical cord blood lead levels. Some environmental variables, such as proximity to traffic-heavy side roads (p = 0.19), presence of greenspace around the house (p = 0.18), visible traffic smoke (p = 0.2), and proximity to gas stations (p = 0.209) were not statistically significant but suggested trends that warrant further exploration in larger and more detailed studies.

### 3.4. Multivariate analysis

Multivariate logistic regression was conducted to assess the association between cord blood lead levels and various characteristics of study participants. Variables entered in the model were chosen based on statistical significance and conceptual relevance based on literature. Then, through an iterative process, variables were retained if they contributed meaningfully to model performance, improved model fit or reflected potential confounders, regardless of statistical significance. Variables with evidence of multicollinearity or poor contribution to model stability were removed. The final model included: age, BMI, gravidity, house location, direct smoking during pregnancy, and proximity to construction sites. Although the house’s location was initially included, it was removed because it was not statistically significant (*p* = 0.179) and did not improve model fit or predictive power. The overall model was statistically significant, with a likelihood ratio chi-squared of 22.81 (*p* = 0.0004), and the pseudo-R-squared value was 0.2578, indicating a moderate fit of the model to the data. The results of the regression analysis are presented in Table 4.

Higher BMI participants were approximately three times more likely to have elevated lead concentrations (OR = 2.94 *p* = 0.005). Participants who continued to smoke during their pregnancy had significantly higher odds of elevated lead levels in their cord blood (OR = 14.94, *p* = 0.035). Finally, participants who resided near construction sites were 87% less likely to have elevated cord blood lead concentrations (OR = 0.13, *p* = 0.037), indicating that living near construction sites was associated with lower lead levels.

## 4. Discussion

This study aimed to assess the cord blood lead levels of participants enrolled in the EELI study, providing a preliminary look at lead exposure in Lebanon among a highly vulnerable sub-population: newborns. While the findings indicate that the vast majority of umbilical cord blood levels are below the Centers for Disease Control and Prevention (CDC) threshold of 3.5 μg/dL, the mere presence of lead in all samples is problematic as no safe level of exposure to lead has been identified, and, even minimal levels, lead can have long-lasting effects on infant development, including cognitive impairments and behavioral problems [12].

In our study, the median cord blood lead concentration was 0.545 μg/dL (mean: 0.719 ± 0.73 μg/dL), with levels ranging between 0.238 μg/dL and 5.44 μg/dL. Our study’s mean concentration was also lower than the pooled mean blood lead concentrations in children from 34 low- and middle-income countries, which ranged between 1.66 and 11.73 μg/dL [13]. These levels are considerably lower than those reported in several other countries across the MENA. Median cord blood BLLs were reported at 2.057 μg/dL in Saudi Arabia [14], 4.9 μg/dL in Morocco [15], 7 μg/dL in Iraq [16], and as high as 11.67 μg/dL in Syria [17]. Compared to studies reporting mean values, our mean of 0.719 μg/dL is also lower than those observed in Libya (6.05 μg/dL) [18], and Turkey (1.69 ± 0.91 μg/dL) [19]. However, our findings align with those reported by Neda et al. [20] in Iran who found the mean of umbilical cord blood lead at 0.65 ± 0.32 μg/dl (0.3–−1.35 μg/dL). In Lebanon, a study by El-Zahran et al. [2] screening for lead in the venous blood of children aged 1–6 years, found a mean concentration of 1.1 ± 0.7 μg/dL. Since our study measures lead concentration in cord blood samples, which reflects lead exposure during fetal development, our results are expectedly lower, reflecting the bio-accumulative nature of lead. It is also worth highlighting that, compared with the high level of blood lead contamination identified by a much earlier study by Nuwayhed et al. in 2003, there is a notable decline in lead exposure in Lebanon. This decline can be attributed to many factors, notably the nationwide ban on leaded gasoline.

In our study, we found that higher pre-pregnancy BMI was associated with elevated cord blood lead concentrations, with mothers having higher BMI being about three times more likely to have elevated levels. While some studies [21] found no effect of maternal weight and BMI on lead concentrations, other studies by Polanska et al. [22], Lewicka et al. [23] and Hernández-Mendoza et al. [24] reported higher blood lead levels in overweight and obese individuals. Lucas et al. [25] suggest that factors like bile secretion and increased caloric intake may enhance lead absorption, leading to higher levels through food. Moreover, lead exposure has been associated with health issues such as obesity due to lead’s effects on the hypothalamic–pituitary–adrenal axis, thyroid hormones, and reproductive hormones [24].

Interestingly, living near construction sites was associated with lower blood lead levels in our study. This finding aligns with El-Zahran et al. [2] who also found lower blood lead levels in children residing near construction areas in Lebanon. One possible explanation is that active construction tends to take place in newer and redeveloping neighborhoods [2], which are often wealthier, better regulated, and less impacted by legacy lead contamination. However, it is important to note that these results diverge from global literature, which generally links proximity to construction and demolition activities to an increase in lead exposure due to the release of contaminated dust [26–28]. That said, not all studies have found an association between these activities and BLL. Bezold et al. [29] reported no significant association between proximity to demolition sites and BLLs among children in Detroit, possibly due to the type and age of the houses demolished and the enhanced dust control measures. Similarly, Branz et al. [30] found that after adjusting for the year the house was built, and neighborhood socioeconomic characteristics, proximity to demolition sites was not associated with elevated BLL in children in St. Louis, Missouri either. Taken together, these mixed findings suggest that contextual factors are critical. In Lebanon, active construction may be more indicative of safer, modern infrastructure, while the greatest risk may lie in older, unrenovated buildings with legacy lead hazards. This underscores the importance of localized assessments of environmental risk when evaluating predictors of lead exposure.

Active smoking during pregnancy was significantly associated with higher odds of elevated cord blood concentrations, consistent with existing literature [31–33] Although active smoking for women is less common in some MENA countries due to prevailing cultural and social norms, tobacco exposure remains a concern. For example, in a Moroccan cohort of pregnant women [15], passive smoking was found to have a positive association with cord blood lead levels. Given that lead exposure can be detrimental to both maternal and fetal health, these findings highlight the importance of public health interventions aimed at reducing tobacco exposure during pregnancy to mitigate environmental lead exposure. Lastly, while multiple sociodemographic variables such as household income, educational attainment, type of employment, and perceived social status were assessed in the bivariate analysis, they were not retained in the final multivariate model due to limited statistical contribution. Nonetheless, we acknowledge the possibility of residual confounding socioeconomic factors may influence both BMI and residential environmental exposures, and their effects on lead levels may not have been fully captured in the current pilot study.

The current study is not without its limitations. First, the sample size is relatively small and limited to a vulnerable subpopulation, which limits the generalizability of the findings to the broader population. Moreover, lead exposure may arise from various sources, some of which are not included in the original study variables. However, despite these limitations, the findings of this study contribute to addressing a critical gap in the literature in the Middle East and North Africa (MENA) region, where lead exposure remains under-characterized. It provides valuable insights into lead among a highly vulnerable population in Lebanon and highlights the ongoing risk of lead exposure even after the ban of leaded gasoline in Lebanon. Moreover, with the changing landscape due to geopolitical challenges in Lebanon, addressing environmental health risks has become an even greater priority. The instability and increase in environmental contamination with harmful chemicals require urgent action to reduce maternal and newborn exposures. This study is part of a larger project that aims to lay the groundwork for potential scalability, enabling the inclusion of a larger, more nationally representative pool of participants and the expansion of the analysis to encompass more family members in the future. To address this issue and protect the health of future generations, we propose the following key recommendations:

1. The Ministry of Public Health (MoPH) in partnership with the World Health Organization, non-governmental organizations, and academic institutions shall conduct a larger screening campaign to include individuals from diverse backgrounds, and socioeconomic statuses, and a larger sample size to ensure a more representative study population. Different biospecimen types (such as venous blood samples or other techniques) should also be considered. This is necessary because the current study’s population does not represent the entire Lebanese population and focuses only on a vulnerable subpopulation: newborns.
2. Non-governmental organizations, in collaboration with the MoPH and WHO, shall raise awareness about the sources and dangers of heavy metals, harmful chemicals exposures, and the risks associated with both active and passive smoking. Special attention should be given to pregnant women and families with young children.
3. The MoPH shall coordinate with the Ministry of Environment (MoE) to set up an ongoing environmental monitoring program. This program should continuously track lead and other heavy metals in various media, including air, water, and soil. The program should also identify the sources of lead exposure at the national level.
4. The MoPH shall establish periodic screening programs for lead exposure in pregnant women and children. Early identification and mitigation of risks are crucial, as lead can bioaccumulate over time and cause severe health effects. Furman et al. (2001) and Mahdi et al. [34] found strong correlations between maternal blood levels and umbilical cord blood levels, showing that venous blood lead levels can be a good biomarker to determine fetal exposure to lead and can be routinely screened.
5. Academic institutions, research institutes, and non-governmental organizations shall initiate long-term studies on the health effects of chemical exposures to better understand their impact over time. Given that lead was present in all tested cord blood samples, infants are not starting from a lead level of zero. This, combined with the bio-accumulative properties of lead, underscores the need of longitudinal studies to better understand their impact. Projects like the EELI study are already addressing these issues and building the foundation for similar research. Expanding such efforts is vital to fully comprehend the long-term health outcomes of chemical exposures.
6. The MoPH, in collaboration with the paint industry, shall update the newly endorsed lead in paint standards to include a numerical limit for lead additives in domestic paints and ensure its enforcement. This builds on the success of the lead ban in gasoline.
7. Healthcare institutions in Lebanon shall establish and sustain laboratory services to perform blood lead analyses for vulnerable populations, ensuring timely and accurate assessments.
8. The MoPH, in collaboration with non-governmental organizations and academic institutions, should prioritize the training of healthcare workers, including obstetricians, pediatricians, midwives, and primary care providers, on the WHO guidelines for the clinical management of lead poisoning. Training should also equip the healthcare personnel to recognize environmental risk factors, (such as smoking, deteriorating housing, proximity to industrial or contaminated sites), screen high-risk individuals, and counsel pregnant women on exposure prevention. Making this training integral to antenatal care can support early detection and intervention. Simultaneously, the MoPH should invest in the establishment of a state-of-the-art poison center capable of supporting screening, treatment, and community outreach.

## Figures and Tables

**Table 1.** Demographic characteristics of study participants.

Age at gestation (*N*=73)			
*Mean* ± *SD*	31.9 ± 3.91	Min=24	Max=42
**Body Mass Index (BMI) (*N*=71)**			
*Underweight*		4 (5.63%)	
*Normal Weight*		45 (63.38%)	
*Overweight*		22 (30.99%)	
**Gravidity (*N*=72)**			
*Primagravida*		21 (29.17%)	
*Multigravida*		51 (70.83%)	
**Women’s Level of Education (*N*=72)**		**Male Spouse Level of Education (*N*=72)**	
*High School Level or Less*	12/72 (16.67%)	*High School Level or Less*	16/72 (22.22%)
*Bachelor’s Degree or its equivalent*	30/72 (41.67%)	*Bachelor’s Degree or its equivalent*	33/72 (46.83%)
*Master’s Degree or its equivalent*	29/72 (40.28%)	*Master’s Degree or its equivalent*	22/72 (30.56%)
*Doctorate Degree or its equivalent*	1/72 (1.39%)	*Doctorate Degree or its equivalent*	1/72 (1.39%)
**Women’s Employment Status (*N*=72)**		**Male Spouse Employment Status (*N*=72)**	
*Unemployed*	17/72 (23.61%)	*Unemployed*	3/72 (4.17%)
*Employed*	55/72 (76.39%)	*Employed*	69/72 (95.83%)
* Full-time*	40/55 (72.73%)	* Full-time*	46/69 (66.67%)
* Part-time*	3/55 (5.45%)	* Part-time*	3/69 (4.35%)
* Self-Employed*	12/55 (21.82%)	* Self-employed*	20/69 (28.99%)
**Total Household Monthly Income in LBP (*N*=71)**			
*Less than $800*		2/72 (2.78%)	
*$800–$1600*		3/72 (4.17%)	
*$1600-$3500*		19/72 (26.39%)	
*$3500 and above*		35/72 (48.61%)	
*Rather not say*		12/72 (16.67%)	
**Main Breadwinner for Household (*N*=72)**			
*Husband*		31/72 (43.06%)	
*Wife*		2/72 (2.78%)	
*Dual-income household (50–50)*		39/72 (54.17%)	
**MacArthur Subjective Social Status Score (*N*=72)**			
*Within Community*			
* Medium*		37 (51.39%)	
* High*		33 (45.83%)	
* Rather not say*		2 (2.78%)	
*Nationally*			
* Low*		10 (13.89%)	
* Medium*		28 (38.89%)	
* High*		32 (44.44%)	
* Rather not say*		2 (2.78%)	

**Table 2.** Characteristics of participants^ǂ^, stratified by their blood lead comparison with the median.

Variable	Total	Below Median^ǂǂ^	Above Median^ǂǂ^	P-value
**Age (*N*=73)**				
*24–29*	21	7	14	0.025*
*30–34*	35	16	19	
*35+*	17	13	4	
**BMI (*N*=71)**				
*Less than 18.5*	4	3	1	0.016*
*Between 18.5 and 24.9*	45	27	18	
*25 and above*	16	4	12	
**Gravidity (*N*=72)**				
*Primigravida*	21	6	15	0.02*
*Multigravida*	51	30	21	
**Mother’s Education Level (*N*=73)**				
*High School Level or Less*	13	7	6	0.512
*Bachelor’s Degree or its equivalent*	30	13	17	
*Master’s Degree or its equivalent*	29	17	12	
*Doctorate Degree or its equivalent*	1	0	1	
**Spouse Education Level (*N*=73)**				
*High School or less*	17	7	10	0.378
*BA/BSc*	33	20	13	
*MA/MSc*	22	10	12	
*PhD*	1	0	1	
**Mother’s Employment Type (*N*=73)**				
*Unemployed*	17	7	10	0.895
*Part-time*	4	2	2	
*Full-time*	40	21	19	
*Self-employed*	12	7	5	
**Spouse Employment Type (*N*=73)**				
*Unemployed*	3	2	1	0.577
*Part-time*	3	2	1	
*Full-time*	46	25	21	
*Self-employed*	21	8	13	
**Mother’s Sector of Employment (*n*=73)**				
*Academia and Education*	7	2	5	0.867
*Commerce and Retail*	12	7	2	
*Construction and Architecture*	6	3	3	
*Creative Arts and Media*	2	1	1	
*Finance and Banking Services*	9	4	5	
*Government and Public Services*	3	2	1	
*Healthcare*	11	7	4	
*Other*	6	4	2	
*Not applicable*	17	7	10	
**Spouse Employment Sector (*N*=72)**				
*Agricultural sector*	1	0	1	0.781
*Commerce and Retail*	10	6	4	
*Construction and Architecture*	9	4	5	
*Creative Arts and Media*	2	1	1	
*Finance and Banking Services*	10	4	6	
*Government and Public Services*	8	5	3	
*Healthcare*	4	1	3	
*Manufacturing*	5	2	3	
*Tech IT*	6	5	1	
*Other*	14	6	8	
*Not applicable*	3	2	1	
**Monthly Household Income (*N*=72)**				
*Less than $800*	3	1	2	0.753
*$800–$1600*	3	2	1	
*$1600-$3500*	20	9	11	
*$3500 and above*	35	20	15	
*Rather not say*	12	5	7	
**Main Breadwinner (*N*=72)**				
*Mother*	2	0	2	0.307
*Spouse*	31	14	17	
*Dual-income household (50–50)*	39	22	17	
**MacArthur Subjective Social Status Score - Within Community (*N*=72)**				
*High*	33	16	17	1.000
*Moderate*	37	19	18	
*Rather Not Say*	3	1	1	
**MacArthur Subjective Social Status Score - Within Country (*N*=72)**				
*High*	32	15	17	0.805
*Moderate*	28	16	12	
*Low*	10	4	6	
*Rather Not Say*	2	1	1	

ǂ In the analysis of the associations between sociodemographic variables and cord blood lead levels, the total number of participants varies and may not always equal the total sample size (*N* = 75) due to incomplete questionnaire responses from some participants.

ǂǂ Participants were stratified by whether their cord blood lead levels were above or below the sample median value (0.545 μg/dL)

**Table 3.** Associations between environmental variables^ǂ^ and cord blood levels, stratified by position according to the median.

Variable	Total	Below Median^ǂǂ^	Above Median^ǂǂ^	p-value
**House Location (*N*=72)**				
*Aley*	5	1	4	0.031*
*Baabda*	12	9	3	
*Beirut*	4	1	3	
*Byblos-Keserwan*	8	2	6	
*Matn*	39	21	18	
*Other*	4	0	4	
**Type of Household (*N*=64)**				
*Apartment*	59	31	28	0.667
*Independent House*	5	2	3	
**Age of the Building (*N*=72)**				
*1999 and before*	14	7	7	0.312
*2000–2009*	6	5	1	
*2010 and after*	38	19	19	
*Unknown*	14	5	9	
**Home Surface Area (*N*=65)**				
*150 m*^*2*^ *and less*	21	9	12	0.317
*151 m*^*2*^ *to 200 m*^*2*^	26	13	13	
*More than 200 m* ^*2*^	18	12	6	
**Traffic Smoke Visible Indoor (*N*=64)**				
*No*	52	29	23	0.208
*Yes*	12	4	8	
**Traffic Smoke Smell Indoor (*N*=64)**				
*No*	53	29	24	0.331
*Yes*	11	4	7	
**Indoor Smoking (*N*=74)**				
*No*	45	22	23	0.812
*Yes*	29	15	14	
**Active smoking during pregnancy (*N*=74)**				
*No*	67	36	31	0.054
*Yes*	7	1	6	
**Greenspace Surrounding house (*N*=64)**				
*No*	15	10	5	0.181
*Yes*	49	23	26	
**Known application of pesticide around the place of residence (*N*=65)**				
*No*	30	16	14	0.878
*Yes*	35	18	17	
**Pesticide Application Frequency in residential surroundings (number of times per year) (*N*=60)**				
*None*	25	13	12	1.00
*1–2 times*	28	14	14	
*3 times and more*	7	4	3	
**Proximity to agricultural lands (*N*=67)**				
*No*	51	27	24	0.837
*Yes*	16	8	8	
**Proximity to farms (*N*=67)**				
*No*	62	32	30	1.000
*Yes*	5	3	2	
**Proximity to highway/main road (*N*=67)**				
*No*	43	24	19	0.433
*Yes*	24	11	13	
**Proximity to traffic-heavy side roads (*N*=67)**				
*No*	45	21	24	0.192
*Yes*	22	14	8	
**Proximity to gas stations (*N*=67)**				
*No*	43	20	23	0.209
*Yes*	24	15	9	
**Proximity to diesel generators (*N*=67)**				
*No*	39	21	18	0.756
*Yes*	28	14	14	
**Proximity to construction site (*N*=67)**				
*No*	53	27	26	0.680
*Yes*	14	8	6	
**Proximity to large car parks (*N*=67)**				
*No*	57	29	28	0.736
*Yes*	10	6	4	
**Proximity to factories (*N*=67)**				
*No*	61	33	28	0.414
*Yes*	6	2	4	

ǂ In the analysis of the associations between environmental variables and cord blood lead levels, the total number of participants varies and may not always equal the total sample size (*N* = 75) due to incomplete questionnaire responses from some participants.

ǂǂ Participants were stratified by whether their cord blood lead levels were above or below the sample median value (0.545 μg/dL)

**Table 4.** Multivariate logistic regression to assess the association between participants’ characteristics and cord blood lead levels.

Variable	Odds Ratio	Std. Error	z-value	p-value	95% Confidence Interval	Direction
Age category	0.4278	0.1262	−1.70	0.088	0.1611–1.1357	↓ BLL with ↑ age
BMI category	2.9401	1.1262	2.82	0.005*	1.3877–6.2291	↑ BLL with ↑ BMI
Gravidity	0.3229	0.2293	−1.59	0.111	0.0803–1.2986	↓ BLL with ↑ gravidity
Active smoking during pregnancy	14.9359	19.1619	2.11	0.035*	1.2083–184.62	↑ BLL with smoking
Proximity to construction sites	0.1305	0.1273	−2.09	0.037*	0.0193–0.8834	↓ BLL with proximity
Constant	14.8733	21.4397	1.87	0.061	0.881–250.841	

## Data Availability

The data and materials that support the findings of the study are available upon reasonable request from the corresponding author, MM.
